# Quantitative peptidomics of endogenous peptides involved in TGF-β1-induced epithelial mesenchymal transition of renal epithelial cells

**DOI:** 10.1038/s41420-017-0001-x

**Published:** 2018-02-06

**Authors:** Rattiyaporn Kanlaya, Visith Thongboonkerd

**Affiliations:** Medical Proteomics Unit, Office for Research and Development, Faculty of Medicine Siriraj Hospital, and Center for Research in Complex Systems Science, Mahidol University, Bangkok, Thailand

## Abstract

TGF-β1 is a key fibrotic factor mediating epithelial mesenchymal transition (EMT) of epithelial cells through various signaling pathways. However, roles of proteolytic cleavage and endogenous peptide dynamics in TGF-β1-induced EMT remain unknown. We therefore performed quantitative peptidomics of TGF-β1-induced EMT in renal tubular epithelial cells. The acquired mesenchymal characteristics were confirmed, including morphological change (from cobblestone-like to fibroblast-like), decreased epithelial marker (ZO-1), and increased mesenchymal marker (vimentin). Quantitative peptidomics using stable isotope labeling revealed significantly altered levels of 70 unique endogenous peptides (derived from internal and C-terminal parts of 39 unique precursor proteins) after EMT induction. Interestingly, the majority of these peptides were derived from non-short-lived proteins, and analysis of P1 position revealed predominance of hydrophobic residues, suggesting that these endogenous peptides were generated mainly from proteasome cleavage. This hypothesis was confirmed by treating the cells with MG132 (a proteasome inhibitor), which provided almost identical endogenous peptide pattern as of the TGF-β1-treated cells. Moreover, validation assay showed marked reduction of proteasome peptidase activity in both TGF-β1-treated and MG132-treated cells. This is the first peptidome dataset that provides several novel aspects of mechanisms for TGF-β1-induced EMT. Our data also suggest that TGF-β1 exerts inhibitory effect against proteasome activity during EMT induction.

## Introduction

Renal interstitial fibrosis is a common feature of chronic kidney disease (CKD). Patients with CKD are suffered from declined renal function as a consequence of renal injury, nephron loss, and reduction of glomerular filtration rate^1,2^. Transforming growth factor-β1 (TGF-β1) is widely accepted as a major mediator involved in renal fibrosis leading to CKD via various mechanisms, including inflammation, suppression of extracellular matrix (ECM) degradation, activation of residential myofibroblasts, and induction of epithelial mesenchymal transition (EMT) that is associated with increased ECM production and accumulation^3,4^. TGF-β1 triggers signal transduction through its Type I and Type II receptors by forming a signaling complex that subsequently regulates many downstream molecules (e.g., ERK1/2 and Smads) to further induce fibrotic factors^3,5^. While many signaling pathways mediated by TGF-β1 have been characterized, very little is known about roles of TGF-β1 and its downstream pathways in regulation of intracellular proteolytic cleavage.

To date, only a few studies have addressed the interplays between cellular protease activities and TGF-β1 signaling. The active serine protease, plasmin, has been reported to be involved in TGF-β1 activation to promote renal interstitial fibrosis in mice with unilateral ureteral obstruction (UUO), whereas plasmin-deficient mice showed only mild degree of interstitial fibrosis^6,7^. Recent study has demonstrated the potential strategy to inhibit renal fibrosis by using a serine protease inhibitor, camostat mesilate, both in vitro and in vivo^8^. In vitro investigation using a rat kidney fibroblast cell line, NRK-49F, treated with TGF-β1 has shown that camostat mesilate could reduce activation of the downstream targets of TGF-β1 signaling, i.e., phosphorylated ERK1/2 and Smad2/3^8^. In vivo study using rat with UUO has also demonstrated that camostat mesilate could suppress TGF-β1-mediated renal fibrosis by a marked reduction of collagen accumulation in the kidney^8^. Another study has revealed that a urinary trypsin inhibitor, ulinastatin, could attenuate renal interstitial fibrosis in UUO rats by affecting the TGF-β1/Smad pathways^9^.

These findings raise a hypothesis that TGF-β1 and its downstream molecules may affect protease/peptidase activities to aggravate their pathological roles during the development of renal fibrosis. However, there was no previous study to analyze endogenous peptides generated during TGF-β1-induced renal fibrosis or EMT. With the current insights that intracellular peptides bear significant biological functions, the present study thus aimed to characterize the endogenous peptides generated from renal tubular epithelial cells under TGF-β1-induced EMT condition by a quantitative peptidomics approach using stable isotopic labeling, followed by bioinformatic prediction and functional validations.

## Results

### Induction of EMT by TGF-β1 in HK-2 cells

**Fig. 1:**
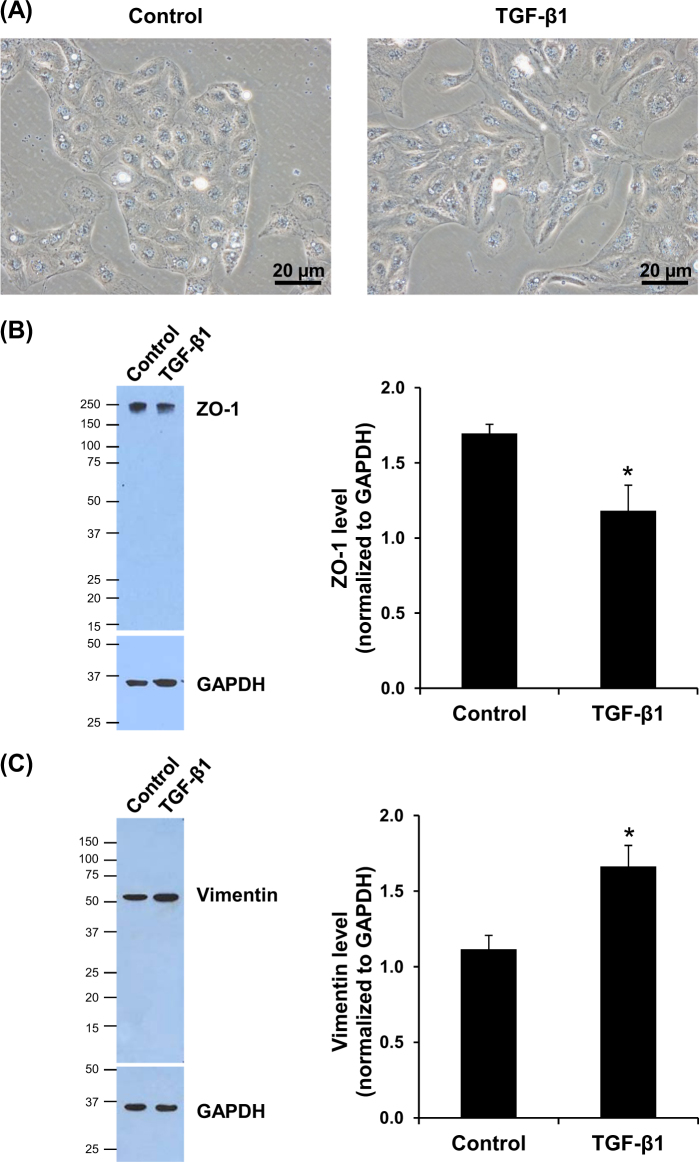
Induction of EMT in HK-2 cells by TGF-β1. **a** Controlled (untreated) cells had cobblestone like morphology, whereas those treated with 5 ng/mL TGF-β1 for 48 h showed fibroblast-like morphology. Original magnification power = ×400. **b, c** Epithelial marker (ZO-1) was significantly decreased, whereas mesenchymal marker (vimentin) was significantly increased. GAPDH served as the protein loading control. Each bar represents mean ± SD of the data derived from three independent experiments. *p < 0.05 vs. control

HK-2 cells were treated with 5 ng/mL TGF-β1 for 48 h and cell morphology was observed under a phase-contrast microscope. The results showed that TGF-β1 induced change of the cell morphology to fibroblast-like, whereas the controlled (untreated) cells still had a typical morphology of epithelial cells (Fig. 1a). In addition, western blotting revealed significant decrease in an epithelial marker ZO-1 (Fig. 1b), whereas the mesenchymal marker vimentin was significantly increased in TGF-β1-treated cells (Fig. 1c).

### Characteristics of the altered endogenous peptides and precursor proteins

Using stable isotopic labeling, we identified a total of 223 individual endogenous peptides in the TGF-β1-treated cells. Quantitative analyses revealed significant changes in levels of 70 unique peptides derived from 39 unique precursor proteins in the TGF-β1-treated cells as compared to the controlled cells (Supplementary Table 1). Most of these altered peptides (64 peptides from 34 precursor proteins) were decreased, whereas six peptides derived from five precursor proteins were increased in the TGF-β1-treated cells. Analysis of the cleavage sites by MEROPs and TopFINDer tools revealed that only 2/5 of the identified altered peptides had known proteolytic enzymes, whereas other 3/5 of them were derived from unknown protease/mechanism (Supplementary Table 1).

**Fig. 2:**
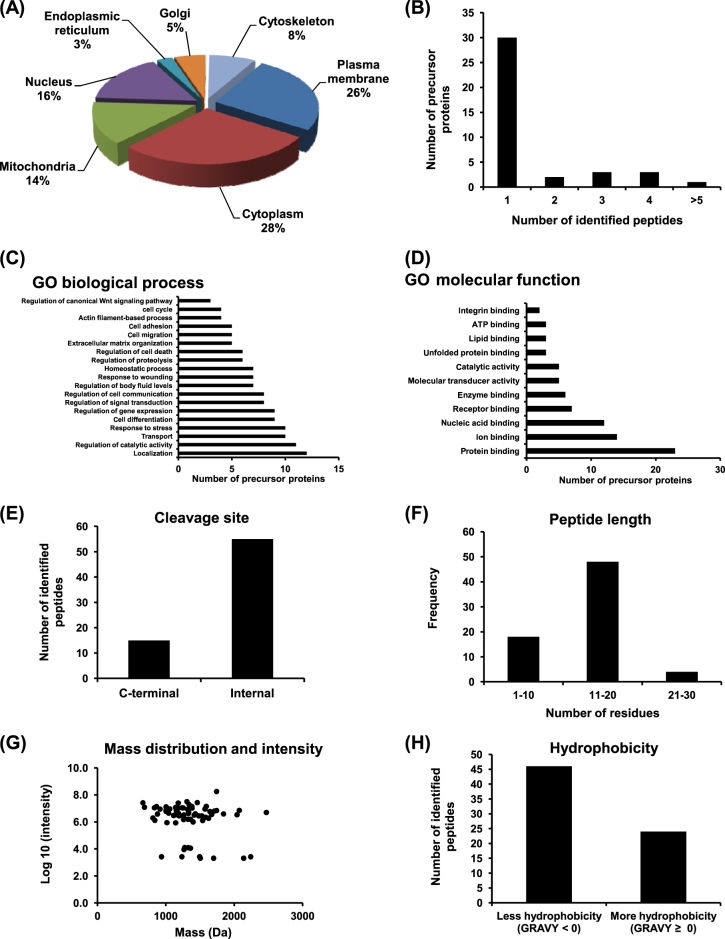
Characteristics of the altered precursor proteins and endogenous peptides in TGF-β1-treated cells. The altered precursor proteins were grouped by their subcellular localization (**a**), number of the identified peptides (**b**), GO enrichment analysis of biological process (**c**), and GO molecular function (**d**). The altered peptides were grouped by their cleavage site (**e**), length (number of amino acid residues) (**f**), mass distribution and intensity (**g**), and hydrophobicity (**h**)

Subcellular localization showed that these altered precursor proteins were localized mainly in cytoplasm (28%), plasma membrane (26%), nucleus (16%), and mitochondria (14%) (Fig. 2a). Frequency of number of the identified peptides from each precursor protein is shown in Fig. 2b. Notably, most of these altered precursor proteins were identified from a single peptide, whereas only one protein was identified with more than five peptides. GO enrichment analysis demonstrated that these altered precursor proteins were involved in many biological processes, e.g., localization, regulation of catalytic activity, transport, response to stress (Fig. 2c). Interestingly, the GO biological processes also included known EMT mechanisms, i.e., cell differentiation, response to wounding, ECM organization, cell migration, actin filament-based process, and regulation of canonical Wnt signaling pathway (Fig. 2c). The enriched GO molecular functions were mainly binding of various cellular molecules, e.g., protein, ion, nucleic acid, receptor, enzyme (Fig. 2d).

The significantly altered endogenous peptides were also characterized for their cleavage sites from the precursor proteins. Most of them were derived from the internal part, whereas some were generated from the C-terminus of the precursor proteins and there were no N-terminus-derived peptides observed (Fig. 2e). Interestingly, more than a half of these altered peptides had 11–20 amino acid residues (Fig. 2f). As expected, all of the altered peptides had molecular mass <3000 Da (most of them were ranged between 1200 and 2000 Da) (Fig. 2g). Grand average of hydropathicity (GRAVY) was measured by Kyte-Doolittle formula to define hydrophobicity of the peptides^10^. The results showed that the GRAVY indices were mostly <0, indicating that most of these peptides were mainly the hydrophilic peptides (Fig. 2h).

### Stability of the altered precursor proteins and endogenous peptides

**Fig. 3:**
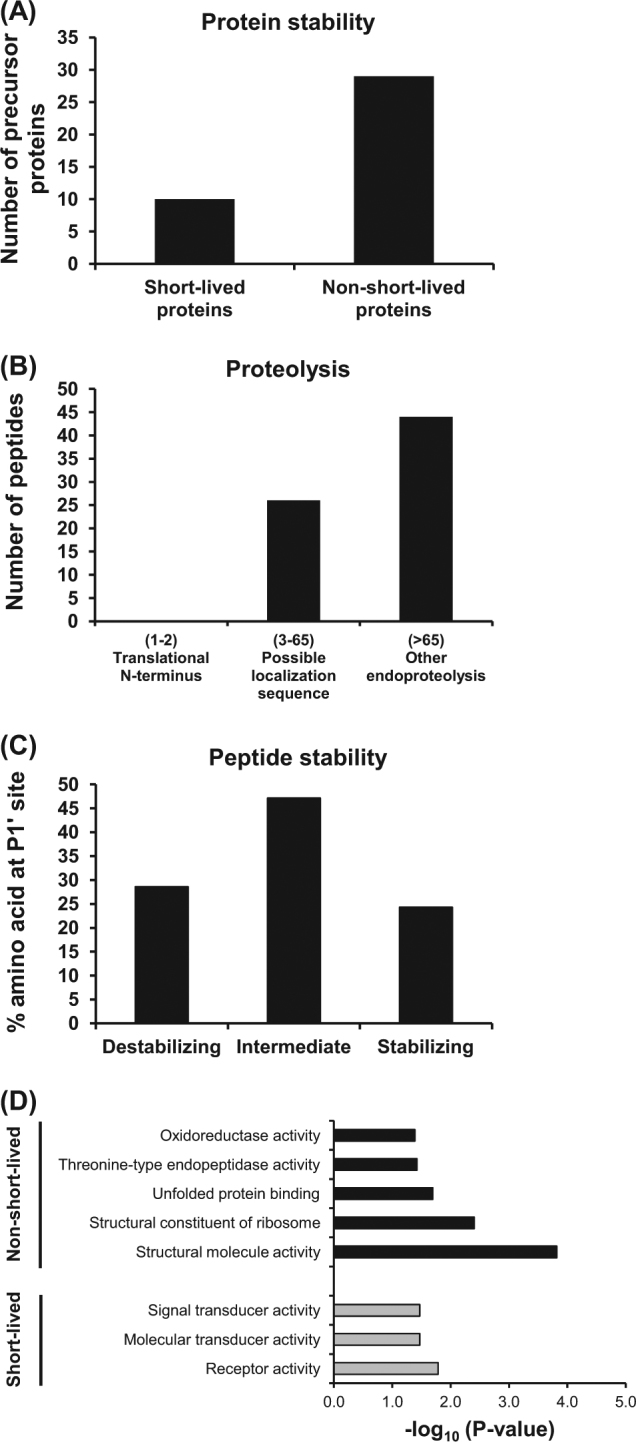
Stability of the altered precursor proteins and endogenous peptides in TGF-β1-treated cells. **a** Number of the precursor proteins based on their stability (short-lived vs. non-short-lived proteins). **b** Grouping of the endogenous peptides according to their three main categories of proteolysis. **c** Peptide stability based on the stability of amino acids at P1′ site. **d** GO enrichment analysis of molecular function of the precursor proteins classified by their stability

Protein stability or half-life should be also accounted for the peptide pool. We thus evaluated half-lives of the precursor proteins by using the SProtP tool. The cutoff lifespans of being classified as short-lived and non-short-lived proteins were <30 and >30 min, respectively. The results showed that most of the precursor proteins were non-short-lived proteins (Fig. 3a). Additionally, the altered peptides were grouped into three main categories of proteolytic process (based on the position of proteolytic cleavage on the precursor proteins), including translational N-terminus, localization sequence (signal or transit peptide removal), and other endoproteolysis processes. The results showed that most of these peptides were derived from other endoproteolysis processes followed by transit peptide removal (Fig. 3b). Interestingly, no any peptide was derived from translational N-terminus processing.

In addition, the peptide stability was analyzed according to the N-end rule pathway, which is one of the important processes for intracellular protein degradation by proteasome. According to the N-end rule pathway, different amino acids at the N-terminus could affect the protein half-life depending on its stability. Amino acid at the P1′ position, which is crucial for the protein half-life, was then analyzed. Interestingly, the intermediate amino acids (S, Y, W, H, A, L, and T with half-life between 1.5 and 20 h) and destabilizing amino acids (Q, R, E, F, D, C, K, and N with half-life <1.5 h) were prominent in these peptides (Fig. 3c). Furthermore, enrichment analysis of molecular function of the precursor proteins based on their half-lives was performed. The statistically significant clusters of molecular functions enriched in non-short-lived proteins included structural molecule activity, structural constituent of ribosome, unfolded protein binding, threonine-type endopeptidase activity, and oxidoreductase activity (Fig. 3d). In contrast, the molecular function enriched in short-lived proteins involved receptor, molecular transducer, and signal transducer activities (Fig. 3d).

Moreover, the endogenous peptides identified in this study were compared to DegraBase, the database of proteolysis in normal and apoptotic human cells^11^. The data showed that only a small number of peptides (nine peptides derived from five different precursor proteins: 10 kDa heat-shock protein, peptidylprolyl *cis*–*trans* isomerase FKBPIA, Peroxiredoxin-5, profilin-1, and vimentin) were overlapped with those deposited in the DegraBase (Supplementary Table 2). All of these peptides were commonly observed in apoptotic cells, whereas some could be found also in healthy cells.

### Analysis of the cleavage sites (P1 and P1‘ positions) of the altered endogenous peptides

**Fig. 4:**
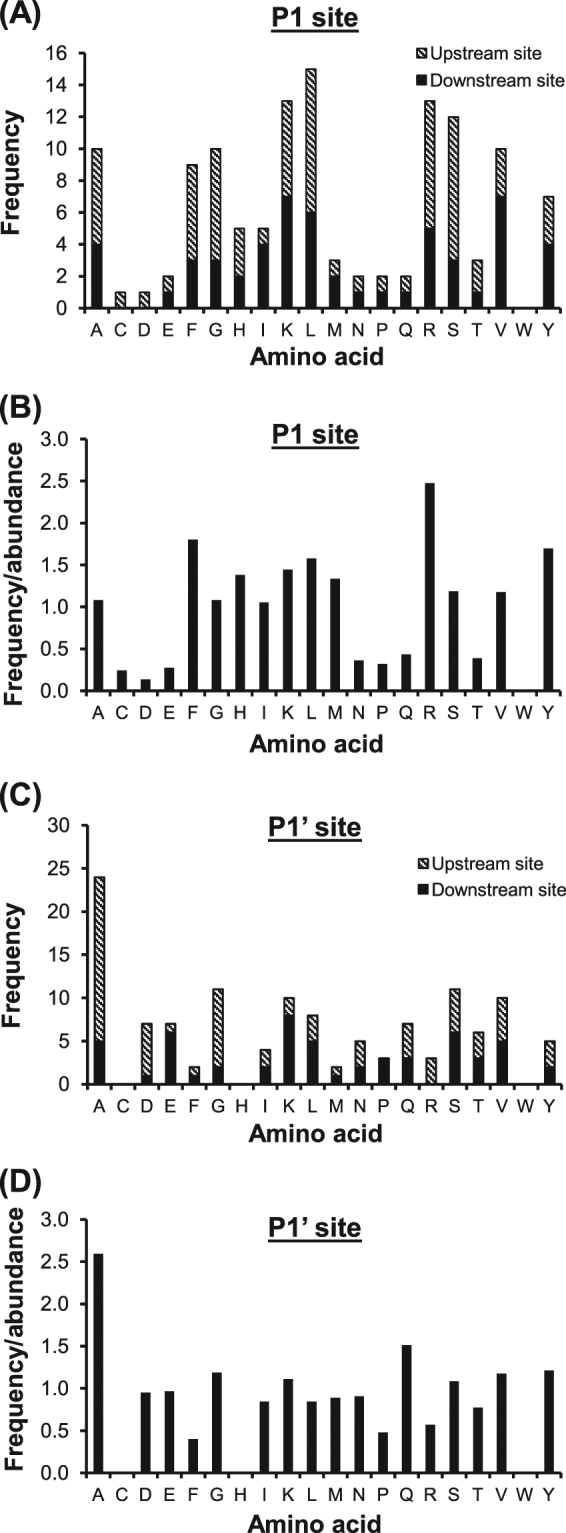
Cleavage sites of the altered endogenous peptides in TGF-β1-treated cells. Frequency and frequency normalized with abundance of each amino acid found at P1 site are illustrated in (**a**) and (**b**), respectively, whereas those for P1′ site are shown in (**c**) and (**d**), respectively

Generally, endogenous peptides require cleavage sites for specific enzyme(s) to recognize and cleave. Internal peptides require both upstream and downstream cleavage sites, whereas N-terminal or C-terminal peptides require only downstream or upstream cleavage site, respectively. According to the general nomenclature of cleavage site positions, P1 and P1′ are defined as the N-terminal and C-terminal sides of the cleaved peptide bond, respectively^12^. Analysis of the frequency of amino acids at P1 position (both upstream and downstream) revealed that leucine, lysine, arginine, and serine were the most common residues found at P1 position (Fig. 4a). Normalization of the amino acid frequency with abundance of each amino acid in human proteins showed that arginine, phenylalanine, tyrosine, and lysine were most prominent at P1 position (Fig. 4b). Similar analysis was performed at P1′ position, the results showed that alanine was mostly found (Fig. 4c). Interestingly, after adjusting with amino acid abundance, alanine remained the most prominent residue found at P1′ position (Fig. 4d).

### Validation of the significant role of proteasome activity to generate endogenous peptides in TGF-β1-treated cells

**Fig. 5:**
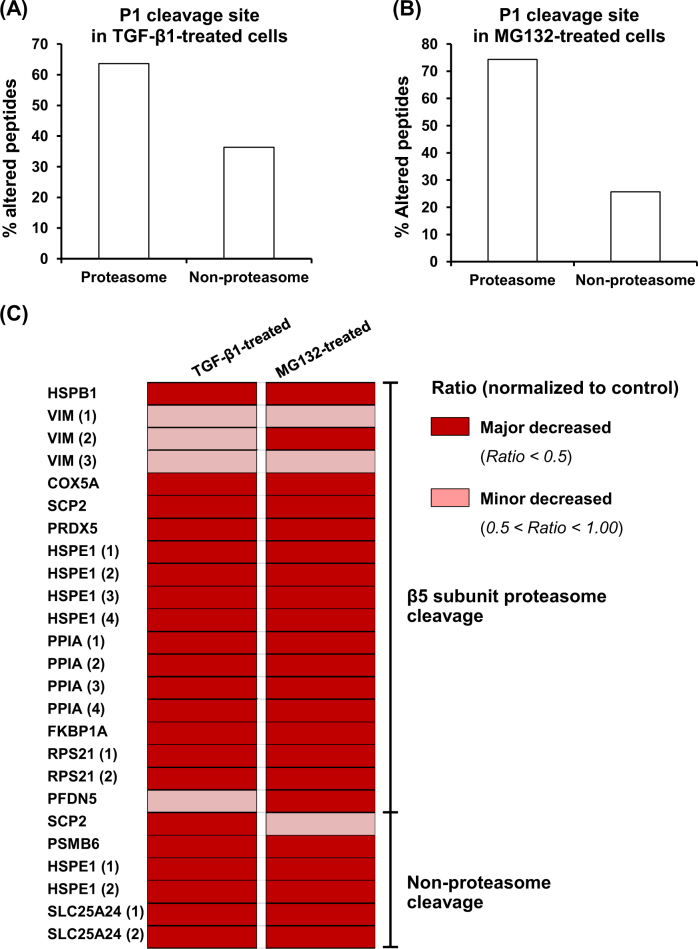
Validation of the significant role of proteasome activity to generate endogenous peptides in TGF-β1-treated cells. **a** Prediction of proteasome-dependent proteolysis of the altered endogenous peptides identified from the TGF-β1-treated cells based on their P1 cleavage site. **b** Experimental confirmation of proteasome-dependent proteolysis of the altered endogenous peptides identified from the proteasome inhibitor (MG132)-treated cells. **c** Heat map analysis of the overlapped endogenous peptides found in both TGF-β1-treated and MG132-treated cells

The proteolytic activity of proteasome depends on its three main subunits, including β1, β2, and β5, which have caspase-like, trypsin-like, and chymotrypsin-like activities, respectively. These subunits face the inner cylinder of 20S-proteasome structure and altogether cleave proteins into small peptides. To address whether the altered endogenous peptides found in TGF-β1-treated cells were derived from proteasome activity, the peptide cleavage sites at P1 position were further analyzed for proteasome cleavage by β1-subunit (at acidic residues: D and E), β2-subunit (at basic residues: H, K and R), and β5-subunit (at hydrophobic residues: A, F, I, L, V, W, and Y). Other amino acids found at P1 position (C, G, M, N, P, Q, S, and T) were grouped as non-proteasome cleavage site. For internal peptides that require two cleavage sites, they were grouped into β1- or β2-cleaved peptides if either side was found with acidic or basic residue, respectively. The β5-cleaved peptides were considered when both sides were found with hydrophobic residues. The results revealed that 64% of the altered endogenous peptides contained the proteasome cleavage sites (Fig. 5a).

To validate such prediction, an additional experiment was performed. Endogenous peptides were extracted from the cells treated with MG132, the proteasome inhibitor, and then analyzed for the amino acid residue at P1 position. The data showed that MG132 caused significant changes in levels of 210 unique peptides derived from 75 unique proteins in MG132-treated cells (Supplementary Table 3). As expected, 74% of the endogenous peptides were derived from proteasome cleavage (Fig. 5b), consistent with the data observed from initial prediction in the TGF-β1-treated cells (Fig. 5a). To further address whether TGF-β1 could inhibit proteasome activity, only the altered peptides that were identified from both TGF-β1- and MG132-treated cells were analyzed. The data showed that levels of all these common altered peptides were decreased when compared to the controlled (untreated) cells and their cleavage sites were attributed mainly from the activity of β5-subunit of proteasome (Fig. 5c). Interestingly, patterns of these altered peptides induced by the two different treatments were almost identical, confirming that TGF-β1 exerted inhibitory effect against proteasome activity during EMT development in renal tubular epithelial cells.

**Fig. 6:**
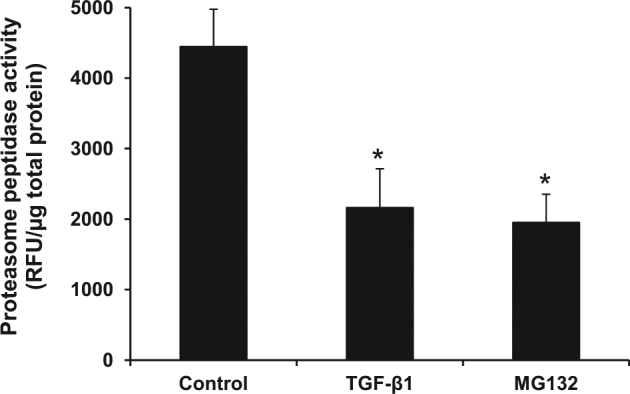
Measurement of proteasome peptidase activity. Proteasome peptidase activities of the controlled (untreated), TGF-β1-treated, and MG132-treated cells were directly measured by an enzymatic assay (see details in Materials and Methods). The peptidase activity is reported as the relative fluorescence unit (RFU) normalized by total protein level. Each bar represents mean ± SD of the data derived from three independent experiments. *p < 0.05 vs. control

Finally, direct measurement of proteasome peptidase activity was performed to further confirm our hypothesis. The data showed that proteasome peptidase activity was marked reduced (by approximately a half) in both TGF-β1-treated and MG132-treated cells as compared to the controlled cells (Fig. 6). And such activity was comparable (almost equal) in TGF-β1-treated and MG132-treated conditions (Fig. 6).

## Discussion

Peptidomics is a comprehensive study of peptide contents (termed as “peptidome”) in a biological sample that may be naturally derived from small coding RNA or intracellular proteolytic cleavage^13,14^. Initially, peptidomics analyses had been conducted mainly for studying peptide hormones or neuropeptides^15,16^. Indeed, naturally occurred endogenous peptides can be found in other tissues and cells^17,18^. Intracellular peptides are postulated to play crucial roles in a vast variety of cellular functions, including regulation of protein–protein interactions^19^ and intracellular signal transduction^20^. Importantly, several studies have revealed clinical impact of intracellular peptides in biomarker discovery for many kidney diseases, e.g., acute kidney injury^21^, acute renal allograft rejection^22^, diabetic nephropathy^23,24^, and CKD^25^.

In the present study, we performed quantitative peptidomics analysis of TGF-β1-induced EMT in renal tubular epithelial cells. When the cells underwent EMT, a set of endogenous peptides were altered in their levels as compared to the controlled or untreated cells. Interestingly, most of the peptides had decreased levels and were derived from non-shorted-lived proteins. Additionally, only a small number of these peptides were generated from known enzymatic cleavage, while most of them were derived from unknown protease/mechanism. However, after analysis of the peptide cleavage sites, the results suggested that both known proteolytic enzymes (e.g., matriptase-2, maprin, and matrix metallopeptidase-2) and unknown proteases found in this study were most likely the members of serine protease, which is the major protease family responsible for protein degradation^26^. In addition to the known biological processes related to EMT, the clusters of precursor proteins involving regulation of catalytic activity and transport were significantly enriched. In concordance with our results, Cha et al.^27^ have previously highlighted the importance of metabolic trade-off between anabolic and catabolic states of cancer cells during EMT in order to survive under metabolic stress. Moreover, recent findings in experimental animals with fibrosis and cultured cells have shown the deregulation of several transporters in fibrotic kidney and TGF-β1-treated HK-2 cells^28^. These findings support that diverse cellular functions, including catabolic process and cellular transport, are deregulated during EMT.

Previous study has revealed the role of proteolytic processing by lysosomal pathway in cancer cell invasion^29^. The data have shown that lysosomal proteins (in particular, cysteine cathepsins) were increased in TGF-β1-induced EMT in mammary cancer cells and facilitated cell invasion. Interestingly, inhibition of cathepsin activity could reduce cell invasion^29^. It is known that proteolytic cleavage by lysosomal pathway is crucial for maintaining cellular homeostasis. In the present study, we also identified the altered peptides that were derived from cathepsin C (also known as dipeptidyl peptidase 1) and cathepsin D. These findings strengthened the important role of lysosomal proteolytic cleavage for regulation of the cellular homeostasis.

Characterizations of the altered peptides induced by TGF-β1 in our present study also revealed that their sizes were ranged from 6 to 30 residues, while the masses were <3000 Da for all the peptides identified. Additionally, proportion of these peptides showed that the peptides with larger size were less abundant. Moreover, analysis of the cleavage site at P1 position showed that the hydrophobic residues were more prominent. These characteristics of the peptides were most likely due to proteasome activity^30^. To decipher the contribution of proteasome to peptide generation in TGF-β1-treated cells, we thus used a proteasome inhibitor (MG132) to inhibit the proteasome activity. MG132 is a peptide aldehyde (carbobenzyl-Leu-Leu-Leu-aldehyde) that can strongly inhibit β1 (caspase-like) and β5 (chymotrypsin-like) subunits of 20S proteasome^31^. The concentration of MG132 used in this study was based on that applied to a previous study demonstrating that 0.5 µM was the lowest concentration of MG132 to inhibit proteasome activity without any effect on viability of HK-2 cells^32^. As expected, the majority of the altered endogenous peptides in the MG132-treated HK-2 cells were decreased, confirming that proteasome played significant role in proteolytic cleavage to generate endogenous peptides in HK-2 renal tubular cells.

The catalytic activity of proteasome, in particular β2 and β5 subunits, has been demonstrated to be suppressed in TGF-β1-induced EMT in breast mammary cancer cells^29^. Additionally, treatment with specific inhibitors of these two subunits was associated with the increase in EMT-related genes/proteins^33^. In concordance, our present study also showed that most of the endogenous peptides extracted from TGF-β1-treated cells were decreased in their levels. The results were consistent when the cells were treated with the proteasome inhibitor MG132. Moreover, levels of all the altered peptides commonly found in both TGF-β1- and MG132-treated cells were decreased. Comparing to the quantitative studies of intracellular peptidome, our data were consistent with the previous findings demonstrating that non-borate inhibitors (including epoxomicin, clasto-lactacystin β-lactone, and MG132) caused markedly decreases of the intracellular peptides^32,34^. Additionally, we found several peptides derived from 40S ribosomal protein S21, cytochrome *C* oxidase subunit 5, peptidylprolyl isomerase A, and peroxiredoxin-5 had significantly decreased levels in both TGF-β1- and MG132-treated cells, consistent with those previously reported in the cells treated with various proteasome inhibitors^32,34^. Collectively, our findings thus confirmed the hypothesis that TGF-β1 exerted inhibitory effect against proteasome activity, resulting in the decreased levels of endogenous peptides produced in TGF-β1-treated cells. One might argue that the treatment itself would cause accumulation of precursor proteins, thereby subsequently reduced intracellular peptide pool. However, previous systematic evaluation of protein degradation by ubiquitin-modified proteome using quantitative proteomics approach has revealed that most of the cellular proteins were not accumulated when the proteasome activity was inhibited^35,36^. Moreover, direct measurement of proteasome peptidase activity in the present study clearly demonstrated the markedly reduced peptidase activity of proteasome in TGF-β1-treated cells as compared to the controlled cells, consistent with the data observed in the MG132-treated condition. Taken together, the decreased peptide levels observed in our present study was most likely resulted from the inhibition of proteasome activity, indicating that the majority of endogenous peptides generated in TGF-β1-treated HK-2 were derived from proteasome cleavage.

In conclusion, we demonstrate herein alterations in peptidome of renal tubular epithelial cells after induction of EMT by TGF-β1. Our data also revealed the role of proteasome in generation of endogenous peptides during the physiologic condition. Moreover, TGF-β1 exerted inhibitory effect on proteasome activity that might be another relevant mechanism of TGF-β1 to induce EMT. The information regarding regulation of proteolytic cleavage can be added to a growing list of molecular mechanisms of renal fibrosis mediated by TGF-β1 and may facilitate the ultimate goal to define effective strategy to prevent CKD.

## Materials and methods

### Cell culture and treatment

A human proximal renal tubular cell line (HK-2) was cultivated in a growth medium containing Dulbecco’s modified Eagle’s medium (DMEM) (Gibco, Grand Island, NY) supplemented with 10% inactivated fetal bovine serum (FBS) (Gibco) in the presence of 100 U/mL penicillin G and 100 mg/mL streptomycin (Sigma; St. Louis, MO). The cells were maintained in a humidified incubator at 37 °C with 5% CO_2_.

To induce EMT, HK-2 cells were seeded at a density of 5 × 10^4^ cells/well in six-well plate and cultured in the growth medium for 24 h. The growth medium was then replaced with serum-free DMEM for 6 h followed by treatment with 5 ng/mL TGF-β1 (BioLegend, San Diego, CA) in a maintenance medium containing DMEM supplemented with 1% FBS for further 48 h. The cells without TGF-β1 treatment served as the control. Changes in cell morphology were observed under a phase-contrast microscope (Olympus CKX41; Olympus Co. Ltd, Tokyo, Japan). The cells were then subjected to endogenous peptide extraction and quantitative peptidomics analysis.

### Western blotting

At the indicated time-point, the cells were harvested and lyzed by Leammli’s buffer. Protein concentration was quantified by Bradford’s method using Bio-Rad Protein Assay (Bio-rad, Milano, Italy). Proteins with an equal amount of 30 µg per each sample were resolved by 12% SDS-PAGE. The resolved protein bands were electrophoretically transferred onto a nitrocellulose membrane and non-specific bindings were blocked by 5% skim milk in phosphate-buffered saline (PBS). The membrane was then probed with rabbit polyclonal anti-ZO-1 (Santa Cruz Biotechnology, Santa Cruz, CA) or mouse monoclonal anti-vimentin (Santa Cruz Biotechnology) antibody (both at a dilution of 1:1000 in 1% skim milk in PBS) at 4 °C overnight. After washing with PBS, the membrane was incubated with horseradish peroxidase-conjugated swine-anti-rabbit IgG or rabbit-anti-mouse IgG secondary antibody (Dako, Denmark A/S, Denmark) (both at a dilution of 1:2000 in 1% skim milk/PBS). Detection of GAPDH was performed to serve as an equal loading control using mouse monoclonal anti-GAPDH (Santa Cruz Biotechnology) (1:1000 in 1% skim milk/PBS) as a primary antibody. The reactive protein bands were visualized by SuperSignal West Pico chemiluminescence substrate (Pierce Biotechnology, Inc., Rockford, IL) and autoradiography. Band intensity was quantitated using ImageMaster 2D Platinum software (GE Healthcare, Uppsala, Sweden).

### Extraction of endogenous peptides and measurement of peptide concentration

The cells were harvested by trypsinization from three independent 100-mm culture dishes per each group. The endogenous peptides were extracted as previously described^37^. Briefly, the cell pellet was collected by centrifugation at 800 *g* for 5 min at 4 °C. Thereafter, the pellet was added with 1 mL hot water (80 °C) and further incubated at 80 °C for 20 min. The sample was centrifuged at 13,000 *g* and 4 °C for 5 min and then kept at −80 °C overnight. After thawing at room temperature (set at 25 °C), the sample was centrifuged at 13,000 *g* and 4 °C for 15 min. The supernatant was collected and sample volume was reduced by a half using a vacuum concentrator (Savant, Holbrook, NY). The remaining supernatant was acidified with ice-cold 0.1 M HCl for 15 min on ice (to make a final concentration of 10 mM HCl). After centrifugation at 13,000 *g* and 4 °C for 40 min, the supernatant containing crude endogenous peptides was further isolated by a Nanosep 10K device (Pall Corporation, Port Washington, NY). The flow-through fraction was collected and dried by using a vacuum concentrator. Peptide concentration was determined by BCA assay (Pierce Biotechnology, Inc.) as described previously^38^.

### Labeling the endogenous peptides with stable isotope

Dimethyl stable isotope labeling was performed using C18 stage tip packed with POROS R3 beads (Applied Biosystems, Waltham, MA). Initially, the C18 stage tips were washed twice with 100 µL acetonitrile (ACN) and equilibrated twice with 100 µL 1% trifluoroacetic acid (TFA). Endogenous peptides were dissolved in 100 µL of 1% TFA and applied to the stage tips, followed by washing with 100 µL of 0.1% TFA twice. The light and heavy labeling solutions were prepared separately. The labeling reagent was prepared in 50 mM sodium phosphate buffer (pH 7.5) containing 50 µL of 4% (v/v) of either CH_2_O (light) (Sigma) or CD_2_O (heavy) (Cambridge Isotope Laboratories, Tewksbury, MA) and 250 µL of 0.6 M sodium cyanoborohydride (NaBH_3_CN) (Sigma). The stage tips were applied five times with 100 µL labeling reagent separately (allowing the reaction for 5 min each time). The peptides extracted from the controlled and TGF-β1-treated cells were separately incubated with the light and heavy labeling solutions, respectively. After labeling, the stage tips were washed twice with 100 µL of 0.1%TFA and the peptides were eluted with 100 µL of 50% ACN in 0.1% TFA followed by 100 µL of 70% ACN in 0.1% TFA. An equal amount of the labeled peptides from each condition was mixed and dried using vacuum concentrator. The peptides were finally dissolved in 0.1% formic acid (FA) and further analyzed by nanoLC-MS/MS.

### NanoLC-MS/MS using LTQ Orbitrap-XL mass spectrometer and data analyses

The peptide mixture was resuspended in 5 µL of 0.1% FA and separated by nanoflow liquid chromatography (nanoLC) using the EASY-nLC system (Thermo Scientific, Waltham, MA) equipped with a pre-column (EASY-Column, 2-cm-long, 100-µm-diameter) packed with 5-µm C18-A1 (Thermo Scientific) connected to an analytical column (EASY-Column, 10-cm-long, 75-µm-diameter) packed with 3-µm C18-A2 (Thermo Scientific). Two-mobile-phase system consisting of buffer A (0.1% FA) and buffer B (95% ACN/0.1% FA) was used. The peptides were separated using a linear gradient of 0–45% buffer B over 160 min at a flow rate of 200 nL/min. The eluted peptides were directly electrosprayed into the LTQ Orbitrap-XL mass spectrometer (Thermo Scientific), which was operated in a collision-induced dissociation top-12 mode under the control of the Xcalibur 2.1.0 and LTQ Tune Plus 2.5.5 software (Thermo Scientific). The cycle of one full scan was performed at a resolution of 30,000 (350–1800 *m/z* mass range) followed by 12 data-dependent MS/MS scans at a resolution of 7500. The normalized collision energy at 47% and the activation time at 10 ms were set for acquiring mass spectra. Labeled peptide samples prepared from three biological replicates per each group were subjected to nanoLC-MS/MS analyses in technical triplicate (a total of nine MS/MS runs were performed for each group).

The resulting files were analyzed by Proteome Discoverer v.1.4 (Thermo Scientific). The workflow included peptide identification and quantification. The SEQUEST algorithm was used to search against Human Swiss-Prot FASTA database (with 20,205 entries as of June 2017). The search parameters included proteolytic enzyme = none; precursor mass tolerance = 10 ppm; and fragment mass tolerance = 0.6 Da. Oxidation of methionine, dimethyl (K), dimethyl (N-term), dimethyl:2H(4) (K), and dimethyl:2H(4) (N-term) were selected as dynamic modifications. The target false discovery rate was set at <1% by performing a decoy database search. SEQUEST parameters were configured as follows: delta cn ≥ 0.05, Xcorr ≥ 2.0 for 2+, ≥2.5 for 3+, and ≥3.0 for >3+ peptide. Precursor ions quantifier processing mode embedded in the Proteome Discoverer was used for quantifying peptides and ratio of the peak intensity of heavy (treated sample) vs. light (controlled sample) labeled corresponding peptide was obtained. Peptides that were detected only in the control are reported as ratio = 0.01, whereas those detected only in the treated sample are reported as ratio = 100.

### Bioinformatics analyses

Annotation of proteolytic cleavage and other relevant information of the identified peptides were acquired using various peptidase/peptidome databases, including MEROPS (http://merops.sanger.ac.uk), TopFINDer (http://clipserve.clip.ubc.ca/topfind/topfinder), DegraBase (https://wellslab.ucsf.edu/degrabase), and SProtP (http://reprod.njmu.edu.cn/sprotp)^11,39–41^. STRING tool (http://string-db.org) was used to classify protein precursors according to their biological processes and molecular functions^42^. ProteinCenter software (Thermo Scientific) was used for classification of subcellular localization of the precursor proteins. Grand average of hydropathicity (GRAVY) index computed by using ProtParam tool (http://web.expasy.org/protparam) was used to determine hydrophobicity of the peptides^10^.

### Proteasome inhibition

To inhibit proteasome activity, the cell monolayer was treated with 0.5 µM MG132 (a proteasome inhibitor) (Tocris Bioscience, Bristol, UK) in the maintenance medium for 8 h prior to endogenous peptide extraction and quantitative peptidomics and bioinformatics analyses as aforementioned.

### Measurement of proteasome peptidase activity

Proteasome peptidase activity was measured using the protocol previously described by Valera et al.^43^ with slight modification. Briefly, after treatment with TGF-β1 or MG132 as aforementioned, the monolayers of controlled (untreated), TGF-β1-treated, and MG132-treated cells were washed with PBS three times, harvested by gentle scrapping, and disrupted with a lysis buffer containing 50 mM HEPES (4-(2-hydroxyethyl)-1-piperazineethanesulfonic acid) (pH 7.8), 10 mM NaCl, 1.5 mM MgCl_2_, 1 mM EDTA (ethylenediaminetetraacetic acid), 1 mM EGTA (ethylenebis(oxyethylenenitrilo)tetraacetic acid), 250 mM sucrose, and 5 mM DTT prior to two cycles of 5-s pulse-on and 5-s pulse-off sonication with 70% amplitude on ice using Sonics Vibra-Cell VCX130 (Sonic & Materials Inc., Newtown, CT, USA). After centrifugation at 16,000 *g* for 10 min at 4 °C, 50 µL of the collected supernatant was added to 200 µL of the assay buffer (containing 2 mM ATP, 50 mM HEPES (pH 7.8), 10 mM NaCl, 1.5 mM MgCl_2_, 1 mM EDTA, 1 mM EGTA, 250 mM sucrose, and 5 mM DTT). Subsequently, a specific substrate (*N*-succinyl-Leu-Leu-Val-Tyr-amino-4-methylcoumarin) (Bachem AG, Bubendorf, Switzerland) was added to the mixture to make its final concentration at 100 µM. The enzymatic reaction was allowed at 37 °C for 25 min and the released fluorescence signals were measured at *λ*360/460 nm (excitation/emission wavelength) using a multi-mode microplate reader (BioTek Instruments, Inc., Winooski, VT). The proteasome peptidase activity was finally reported as relative fluorescence unit (RFU) normalized by total protein level as determined by the Bio-Rad Protein Assay (Bio-Rad, Milano, Italy).

### Statistical analysis

Quantitative data are reported as mean ± SD, unless stated otherwise. Mean difference between two groups was analyzed by Student’s *T*-test, whereas multiple comparisons were performed by one-way analysis of variance (ANOVA) with Tukey’s post-hoc test using SPSS (version 11.5). Statistical significance was considered at a *p*-value less than 0.05.

## Electronic supplementary material

Supplementary Tables S1–S3
