# Supplementary material for: Quantitative peptidomics of endogenous peptides involved in TGF-β1-induced epithelial mesenchymal transition of renal epithelial cells
Source: Cell Death Discov. 2018 Feb 6;4:9. doi: 10.1038/s41420-017-0001-x (PMC5841401; doi:10.1038/s41420-017-0001-x)
Supplement: Supplementary file 1 — Supplementary Tables S1–S3 [file 41420_2017_1_MOESM1_ESM.pdf]

SUPPLEMENTARY TABLES S1-S3

Quantitative peptidomics of endogenous peptides involved in TGF-β1-induced epithelial mesenchymal transition of renal epithelial cells

*Rattiyaporn Kanlaya and Visith Thongboonkerd**
**Correspondence to: thongboonkerd@dr.com (or) vthongbo@yahoo.com*

Supplementary Table S1. Summary of unique endogenous peptides that were significantly altered in TGF-β1-treated HK-2 cells.

Accession	Precursor protein	Peptide	Ratio[a] (TGF-β1 /Control)	Cleavage site	Responsible proteolytic enzyme(s) reported in MEROPs[b] and TopFINDer[c]
Peptides with significantly decreased levels in TGF-β1-treated HK-2 cells (in alphabetical order)					
P61604	10 kDa heat shock protein, mitochondrial	AAETVTKGGIMLPEKSQGK	0.03	Internal	Matriptase-2/meprin beta subunit
		AAETVTKGGIMLPEKSQGKVLQA	0.01	Internal	Matriptase-2/meprin beta subunit
		AVGSGSKGKGGEIQPVSV	0.26	Internal	Cathepsin S/metrix metallopeptidase-2
		TVVAVGSGSKGKGGEIQPVSV	0.21	Internal	Meprin beta subunit/matrix metallopeptidase-2
		QATVVAVGSGSKGKGGEIQPVSV	0.20	Internal	Meprin beta subunit/matrix metallopeptidase-2
		DGDILGKYVD	0.16	C-terminal	Unknown protease
		VGSGSKGKGGEIQPVSV	0.01	Internal	Cathepsin S/matrix metallopeptidase-2
		RDGDILGKYVD	0.06	C-terminal	Unknown protease
		AAETVTKGGIML	0.39	Internal	Matriptase-2/peptidyl-Lys metallopeptidase
		KVGDKVLLPE	0.14	Internal	Matrix metallopeptidase-2/glutamyl peptidase 1
		AAETVTKG	0.27	Internal	Matriptase-2/no report
		AETVTKGGIML	0.28	Internal	Matriptase-2/peptidyl-Lys metallopeptidase
		GSGSKGKGGEIQPVSV	0.33	Internal	Cathepsin S/matrix metallopeptidase-2
		AAETVTKGGI	0.41	Internal	Matriptase-2/no report
		SAAETVTKGGIML	0.64	Internal	Matriptase-2/peptidyl-Lys metallopeptidase?
Q9NWU5	39S ribosomal protein L22, mitochondrial	LVEGPPPPPEPPKTA	0.54	Internal	No information about this protein
P63220	40S ribosomal protein S21	MGESDDSILRL	0.01	Internal	Unknown protease
		AKADGIVSKNF	0.22	C-terminal	Human airway trypsin-like peptidase, DESC1 peptidase, thrombin
		DGIVSKNF	0.27	C-terminal	Human airway trypsin-like peptidase, DESC1 peptidase, thrombin
		ADGIVSKNF	0.26	C-terminal	Human airway trypsin-like peptidase, DESC1 peptidase, thrombin

P62899	60S ribosomal protein L31	YVPVTTFKNLQTVNVDEN	0.01	C-terminal	Unknown protease
P63261	Actin, cytoplasmic 2	FAGDDAPR	0.41	Internal	Unknown protease/trypsin1
P27144	Adenylate kinase 4, mitochondrial	IAQNFGLQH	0.01	Internal	Unknown protease
P18825	Alpha-2C adrenergic receptor	VKATLV	0.50	Internal	No information about this protein
Q6NUK1	Calcium-binding mitochondrial carrier protein SCaMC-1	QIASYPLALVR	0.10	Internal	No information about this protein
		QLASYPLALVR	0.01	Internal	No information about this protein
		IISKEGIPGLYRG	0.12	Internal	No information about this protein
		VVYENMKQTLG	0.50	Internal	No information about this protein
P07339	Cathepsin D	GEATKQPGITGI	0.53	Internal	Unknown protease
		RDPDAQPGGELM	0.41	Internal	Unknown protease
		VGGSVEDLIAKGPV	0.01	Internal	Unknown protease
Q9BXS0	Collagen alpha-1(XXV) chain	AGKGGGR	0.01	Internal	Unknown protease
P78310	Coxsackievirus and adenovirus receptor	AIPVMIPAQSKDGSIV	0.09	C-terminal	No information about this protein
P14927	Cytochrome b-c1 complex subunit 7	AAGFNK	0.21	Internal	No information about this protein
P20674	Cytochrome c oxidase subunit 5A, mitochondrial	GISTPEELGLDKV	0.25	C-terminal	Glutamyl peptidase 1
P53634	Dipeptidyl peptidase 1	DPFNPFELTNH	0.01	Internal	Cathepsin L/unknown protease
P30040	Endoplasmic reticulum resident protein 29	ALPLDTVTFYK	0.06	Internal	Unknown protease
		AGEFIRASGVEARQA	0.30	Internal	RC1339 q.p. (*Rickettsia conorii*)
P04792	Heat shock protein beta-1	TLPPGVDPTQVSSSL	0.35	Internal	Unknown protease
Q9NXK6	Membrane progestin receptor gamma	YGAVNLFSLGSAIAYSA	0.01	Internal	No information about this protein
P22307	Non-specific lipid-transfer protein	QNLQLQPGNAKL	0.14	C-terminal	Unknown protease
		NLQLQPGNAKL	0.01	C-terminal	Unknown protease
		LQNLQLQPGNAKL	0.20	C-terminal	Unknown protease
P04181	Ornithine aminotransferase, mitochondrial	ALEVLEEENLAENADK	0.36	Internal	Unknown protease
O00151	PDZ and LIM domain protein 1	SPASSTTARVITNQY	0.01	Internal	Unknown protease
P62937	Peptidyl-prolyl cis-trans isomerase A	DIAVDGEPLGRVSF	0.02	Internal	MAM peptidase,cathepsinS/L/meprin alpha subunit
		AVDGEPLGRVSF	0.16	Internal	Cathepsin S/L/meprin alpha subunit
		STGEKGFGY	0.24	Internal	Unknown protease
		KHTGPGILSM	0.41	Internal	peptidyl-Lys metallopeptidase/unknown protease
P62942	Peptidyl-prolyl cis-trans isomerase FKBP1A	DVELLKLE	0.37	C-terminal	Unknown protease
P30044	Peroxiredoxin-5, mitochondrial	APIKVGDAIPAVEVF	0.01	Internal	M24 homologue (unknown peptidase)

Q8WW12	PEST proteolytic signal-containing nuclear protein	LGNVHDQDN	0.01	C-terminal	Unknown protease
Q9Y4D7	Plexin-D1	DDNILKI	0.28	Internal	No information about this protein
Q99471	Prefoldin subunit 5	TALGAAQATAKA	0.63	C-terminal	Matrix metallopeptidase-2
P07737	Profilin-1	STGGAPTFNVTVTKT	0.35	Internal	Trypsin1/trypsin1
P28072	Proteasome subunit beta type-6	RSGSAADTQAVADAVTY	0.51	Internal	Unknown protease
Q99436	Proteasome subunit beta type-7	GGVDVTGPHLY	0.53	Internal	Cathepsin L
Q9NY59	Sphingomyelin phosphodiesterase 3	NKCNDDALASKGALFLKVQ	0.01	Internal	No information about this protein
P04179	Superoxide dismutase [Mn], mitochondrial	GDVTAQIALQPALK	0.01	Internal	No information about this protein
P31431	Syndecan-4	GSGSQVPTEPK	0.01	Internal	Unknown protease
P08670	Vimentin	GGPGTASRPSS	0.58	Internal	Unknown protease
		ASSPGGVYATRS	0.59	Internal	Trypsin1/matrix metallopeptidase
		GGPGTASRPS	0.55	Internal	Unknown protease
Q5SVQ8	Zinc finger and BTB domain-containing protein 41	SHEILSP	0.01	Internal	No information about this protein
Peptides with significantly increased levels in TGF-β1-treated HK-2 cells (in alphabetical order)					
Q9H251	Cadherin-23	ATVLFLNATDLDRSREYGQESIIYSL	100.00	Internal	No information about this protein
O14493	Claudin-4	YSAARSAAASNYV	1.44	C-terminal	No information about this protein
P02751	Fibronectin	ETPSQPNSHPI	2.66	Internal	Matrixmetallopeptidase-9,-3,-12,-8
		VRPSQGGEAPR	3.89	Internal	Unknown protease
P08729	Keratin, type II cytoskeletal 7	QEESEQIKTLN	5.07	Internal	Unknown protease
Q4W5G0	Tigger transposable element-derived protein 2	GIPKAAGK	100.00	Internal	No information about this protein

[a] Ratio = 0.01 represents the peptides that were absent in the TGF-β1-treated cells, whereas Ratio = 100 denotes newly presented peptides in the TGF-β1-treated cells.
[b] The database of proteolytic enzymes, substrates, and inhibitors.
[c] The database for characterizes and annotates proteomics-derived N- or C-termini sets for their origin, sequence context and implications for protein structure and function.

Supplementary Table S2. Summary of the identified endogenous peptides that were also found in DagraBase database.

Accession	Precursor protein	Start residue	Peptide	Conditions		
				**Healthy cells**	**Apoptotic cells**	**This study (EMT)**
P61604	10 kDa heat shock protein, mitochondrial	21	SAAETVTK	✓	✓	✓
P61604	10 kDa heat shock protein, mitochondrial	21	SAAETVTKGGIML	✓	✓	✓
P61604	10 kDa heat shock protein, mitochondrial	45	TVVAVGSGSKGKGGEIQPVSV	✓	✓	✓
P61604	10 kDa heat shock protein, mitochondrial	48	AVGSGSKGKGGEIQPVSV	✓	✓	✓
P62942	Peptidyl-prolyl cis-trans isomerase FKBP1A	101	DVELLKLE	ND	✓	✓
P30044	Peroxiredoxin-5, mitochondrial	54	APIKVGDAIPAVEVF	✓	✓	✓
P07737	Profilin-1	91	STGGAPTFNVTVTKT	ND	✓	✓
P08670	Vimentin	16	GGPGTASRPS	ND	✓	✓
P08670	Vimentin	16	GGPGTASRPSS	ND	✓	✓

✓ = Detectable or found.
ND = Not determined.

Supplementary Table S3. Summary of unique endogenous peptides that were significantly altered in MG132-treated HK-2 cells.

Accession	Precursor protein	Peptide	Ratio[a] (MG132/ Control)
Peptides with significantly decreased levels in MG132-treated HK-2 cells (in alphabetical order)			
P61604	10 kDa heat shock protein, mitochondrial	AAETVTKGGIML	0.14
		AAETVTKGGIMLPEKSQGKVLQA	0.24
		RDGDILGK	0.34
		SAAETVTKGGIMLPEKSQGKVLQA	0.39
		AVGSGSKGKGGEIQPVSV	0.41
		QATVVAVGSGSKGKGGEIQPVSV	0.41
		TVVAVGSGSKGKGGEIQPVSV	0.42
		RDGDILGKYVD	0.44
		TVVAVGSGSKGKGGEIQPVSVKV	0.52
		DGDILGKYVD	0.89
		SAAETVTK	0.89
P62333	26S protease regulatory subunit 10B	HAGPITK	0.62
P62191	26S protease regulatory subunit 4	VGSELIQKY	0.17
		GSELIQKY	0.44
P62195	26S protease regulatory subunit 8	EIDSIGSSRL	0.30
Q9Y399	28S ribosomal protein S2, mitochondrial	EPGDQGPAHPPG	0.47
P82650	28S ribosomal protein S22, mitochondrial	AESGSPETKKPT	0.33
		SSEAAESGSPETKKPT	0.83
Q9NWU5	39S ribosomal protein L22, mitochondrial	VEGPPPPPEPPKTA	0.69
P63220	40S ribosomal protein S21	MGESDDSILRL	0.02
		GESDDSILRL	0.17
		AKADGIVSKNF	0.18
		DGIVSKNF	0.35
		KADGIVSKNF	0.59
		ADGIVSKNF	0.63
P62899	60S ribosomal protein L31	KNLQTVNVDEN	0.37
P62917	60S ribosomal protein L8	GTKTVQEKEN	0.31
Q8IZT6	Abnormal spindle-like microcephaly-associated protein	IVIQKY	0.15
P60709	Actin, cytoplasmic 1	ALPHAILRL	0.02
		ALPHALL	0.23
		ALPHAIL	0.28
		EAPLNPK	0.41
		DESGPSIVHR	0.43
		VAPEEHPV	0.50
		ISKQEYDESGPSIVHR	0.57
		ISKQEYDESGPSIVH	0.82
Q8IZF6	Adhesion G-protein coupled receptor G4	THFGVL	0.67
O75947	ATP synthase subunit d, mitochondrial	AEIIPQNQKAIASSL	0.65
P56381	ATP synthase subunit epsilon, mitochondrial	ANAEKTSGSNVK	0.32
P61769	Beta-2-microglobulin	IQRTPKIQVY	0.40
P55285	Cadherin-6	LIHPTGLSTGAL	0.36
		LIHPTGLSTGALV	0.47
Q6NUK1	Calcium-binding mitochondrial carrier protein SCaMC-1	QIASYPLALVR	0.10
		LASYPLALVR	0.26
		QLASYPLALVR	0.33
		IASYPLALVR	0.35
		ASYPLALVR	0.67

		IISKEGIPGLYRG	0.71
P07858	Cathepsin B	IESEVVAGIPRTD	0.12
P07339	Cathepsin D	SRDPDAQPGGELM	0.01
		SRDPDAQPGGELML	0.01
		VFGEATKQPGITF	0.01
		DNNRVGFAEAAR	0.04
		YTVFDR	0.04
		YTVFDRD	0.06
		FDRDNNRVGFAEAAR	0.08
		EGPIPEVLK	0.09
		NNRVGFAEAAR	0.10
		GGVKVERQ	0.12
		NNRVGFAEAA	0.12
		TVFDRD	0.12
		GPIPEVLK	0.19
Q96M83	Coiled-coil domain-containing protein 7	RFEELKNRLKQRSKSSVKVMLSK	0.03
P42771	Cyclin-dependent kinase inhibitor 2A	ARIDAAEGPSDIPD	0.02
P20674	Cytochrome c oxidase subunit 5A, mitochondrial	GISTPEELGLDKV	0.44
P10606	Cytochrome c oxidase subunit 5B, mitochondrial	ASGGGVPTDEEQATGLE	0.64
		GGVPTDEEQATGLE	0.83
P12074	Cytochrome c oxidase subunit 6A1, mitochondrial	HNPHVNPLPTGYEDE	0.78
P53634	Dipeptidyl peptidase 1	AVAATPIPKL	0.32
		DPFNPFELTNH	0.48
		FNPFELTNH	0.50
Q9NS91	E3 ubiquitin-protein ligase RAD18	EAKRPEPPSTSTL	0.59
P24534	Elongation factor 1-beta	GFGDLKSPAGLQVL	0.01
		GFGDLKSPAGLQV	0.17
P84090	Enhancer of rudimentary homolog	LRRQAQQAGK	0.49
P29317	Ephrin type-A receptor 2	LTQEGQGAGSKVHEFQT	0.81
P41567	Eukaryotic translation initiation factor 1	AKDDQLKVHGF	0.09
Q15056	Eukaryotic translation initiation factor 4H	ANPNSAIFGGARPREE	0.23
		ATPLNQVANPNSAIFGGARPREE	0.33
		VVQKEQE	0.59
P63241	Eukaryotic translation initiation factor 5A-1	AVAIKAMAK	0.12
		SAMTEEAAVAIKAMAK	0.13
Q93063	Exostosin-2	ESVLVLDK	0.20
P09382	Galectin-1	GEVAPDAKSFVLNL	0.02
		GEVAPDAKSFVL	0.32
O94925	Glutaminase kidney isoform, mitochondrial	AAPGPKDGPGETDA	0.72
P50440	Glycine amidinotransferase, mitochondrial	AADFIRA	0.47
P04792	Heat shock protein beta-1	DVNHFAPDEL	0.02
		GGSSWPGYVR	0.17
		RVSLDVNHF	0.19
		LGGSSWPGYVR	0.22
		ESRAQLGGPEAAKSDETAAK	0.23
		SRALSRQL	0.24
		QLGGPEAAKSDETAAK	0.26
		TLPPGVDPTQVSSSL	0.28
		SLDVNHF	0.36
		SSGVSEIRH	0.46
		SSGVSEIRHT	0.48
		LGGPEAAKSDETAAK	0.59
P05787	Keratin, type II cytoskeletal 8	ESSDVLPK	0.67

P10619	Lysosomal protective protein	FVESQKDPENSPVVL	0.01
		YGDSGEQIAGFVKE	0.02
		YGDSGEQIAGFVK	0.03
		APDQDEIQRLPGLA	0.03
		DQDEIQRLPG	0.10
		DQDEIQRLPGLA	0.13
		DSGEQIAGFVKE	0.28
		DQDEIQRLPGLAK	0.77
P35579	Myosin-9	GDLPFVVPR	0.30
Q9UI09	NADH dehydrogenase [ubiquinone] 1 alpha subcomplex subunit 12	TDDPPTTKPLTA	0.71
Q16795	NADH dehydrogenase [ubiquinone] 1 alpha subcomplex subunit 9, mitochondrial	AEIEDVKPA	0.77
P22307	Non-specific lipid-transfer protein	QLQPGNAKL	0.39
		QNLQLQPGNAKL	0.40
		LQNLQLQPGNAKL	0.67
		NLQLQPGNAKL	0.70
Q9Y5B8	Nucleoside diphosphate kinase 7	EGILGK	0.67
P50897	Palmitoyl-protein thioesterase 1	LATEGDHLQ	0.07
Q96JY6	PDZ and LIM domain protein 2	YSAPATLSSRA	0.50
P62937	Peptidyl-prolyl cis-trans isomerase A	DIAVDGEPLGRVSF	0.01
		EDENFILKHTGPGILSM	0.01
		ELFADKVPKTAENF	0.02
		ELFADKVPKTAEN	0.07
		AVDGEPLGRVSF	0.12
		ANAGPNTNGSQFF	0.13
		ANAGPNTNGSQF	0.16
		DGEPLGRVSF	0.17
		EDENFILKH	0.18
		VDGEPLGRVSF	0.22
		KHTGPGLLSM	0.26
		KHTGPGILSM	0.27
		STGEKGFGY	0.29
P62942	Peptidyl-prolyl cis-trans isomerase FKBP1A	DVELLKLE	0.17
P30044	Peroxiredoxin-5, mitochondrial	IKVGDAIPAVEVF	0.01
		APIKVGDAIPAVEVF	0.04
		EGEPGNKVNL	0.22
P30086	Phosphatidylethanolamine-binding protein 1	AGAAVDELGKVLTPTQV	0.01
O00625	Pirin	TAGRGIL	0.35
O60828	Polyglutamine-binding protein 1	ANAEASRTKQQD	0.78
P26599	Polypyrimidine tract-binding protein 1	RIIVENL	0.37
		RLIVENL	0.39
Q99471	Prefoldin subunit 5	TALGAAQATAKA	0.25
P07737	Profilin-1	STGGAPTFNVTVTKT	0.02
Q15185	Prostaglandin E synthase 3	VEDSKDVNVNF	0.02
P28072	Proteasome subunit beta type-6	RSGSAADTQAVADAVTY	0.31
Q99497	Protein DJ-1	AGPTALLAHEI	0.13
P60903	Protein S100-A10	QKDPLAVDKI	0.24
Q7Z7A4	PX domain-containing protein kinase-like protein	AKPKDPFLKKYCNPKKIQ	0.01
P98179	RNA-binding protein 3	NTDEQALEDHF	0.10
		TNPEHASVAMR	0.23
P42345	Serine/threonine-protein kinase mTOR	IGDGLVKP	0.37
P63313	Thymosin beta-10	NTLPTKETIEQEKRSEIS	0.09
		ETQEKNTLPTKETIEQEKRSEIS	0.19

P62328	Thymosin beta-4	TETQEKNPLPSKETIEQEKQAGES	0.01
		NPLPSKETIEQEKQAGES	0.19
		TQEKNPLPSKETIEQEKQAGES	0.29
		ETQEKNPLPSKETIEQEKQAGES	0.36
Q15654	Thyroid receptor-interacting protein 6	TTASTPAGPAFPVQVKV	0.04
P60174	Triosephosphate isomerase	LVGGASLKPEFVD	0.01
		SLGELIGTLNA	0.35
		LDPKIAVA	0.62
Q14157	Ubiquitin-associated protein 2-like	VAPTSSKQH	0.18
Q6EMK4	Vasorin	VTQAREGNLPLLIAPALAA	0.05
P08670	Vimentin	GPGTASRPSSSR	0.05
		SSPGGVYATRS	0.09
		ASSPGGVYATRS	0.11
		GPGTASRPSSS	0.11
		GPGTASRPSS	0.13
		SLGSALRPSTSRSL	0.18
		QAQIQEQHV	0.21
		YASSPGGVYATRSS	0.21
		SLGSALRPSTSRSLY	0.23
		SSPGGVYATRSSA	0.25
		ASSPGGVYATRSS	0.26
		YVTTSTRTY	0.26
		YASSPGGVYATRS	0.26
		RSSVPGVRL	0.30
		RSSVPGVRLL	0.30
		TVETRDGQVINETSQHHDDLE	0.31
		FGGPGTASRPSSSRS	0.31
		SSPGGVYATRSS	0.31
		SPGGVYATRSS	0.31
		ASSPGGVYATRSSAVRL	0.33
		ANYLDKV	0.34
		TRSSAVRL	0.35
		ANYIDKV	0.36
		QVINETSQHHDDLE	0.40
		FGGPGTASRP	0.44
		FGGPGTASRPS	0.45
		ASSPGGVYATR	0.45
		YVTTSTRTYS	0.47
		GGPGTASRP	0.51
		SSVPGVRL	0.57
		SSVPGVRLL	0.57
		RDGQVINETSQHHDDLE	0.60
		MFGGPGTASRPSSS	0.62
		GGPGTASRPS	0.62
		FGGPGTASRPSS	0.65
		YASSPGGVYATR	0.65
		GGPGTASRPSS	0.69
		SLGSALRPSTSR	0.71
		SYVTTSTRTY	0.83
		SLYASSPGGVYATR	0.83
		VETRDGQVINETSQHHDDLE	0.88
Q8N1W2	Zinc finger protein 710	QLDRLDI	0.19

Peptides with significantly increased levels in MG132-treated HK-2 cells (in alphabetical order)			
Q96EL3	39S ribosomal protein L53, mitochondrial	GDKPGADTGR	1.22
P04792	Heat shock protein beta-1	SPAVAAPAYSR	1.37
Q8WXG6	MAP kinase-activating death domain protein	HIKKCNTVRGVFVLEEFVPEIKEV	17.33
Q9NX14	NADH dehydrogenase [ubiquinone] 1 beta subcomplex subunit 11, mitochondrial	FDPSKIQLPEDE	3.33
Q12816	Trophinin	SKGKKAASRGPNSVSEISEAPLATQIV	39.61
P08670	Vimentin	GGPGTASRPSSS	1.33

[a] Ratio = 0.01 represents the peptides that were absent in the TGF-β1-treated cells, whereas Ratio = 100 denotes newly presented peptides in the TGF-β1-treated cells.
